# Predicting Pattern Standard Deviation in Glaucoma: A Machine Learning Approach Leveraging Clinical Data

**DOI:** 10.3390/vision9030077

**Published:** 2025-09-01

**Authors:** Raheem Remtulla, Patrik Abdelnour, Daniel R. Chow, Andres C. Ramos, Guillermo Rocha, Paul Harasymowycz

**Affiliations:** 1 Department of Ophthalmology & Visual Sciences, McGill University, Montreal, QC H4A 0A4, Canada; raheem.remtulla@mail.mcgill.ca (R.R.); andres.ramos@mcgill.ca (A.C.R.); guillermo.rocha@mcgill.ca (G.R.); 2 Faculty of Medicine and Health Sciences, McGill University, Montreal, QC H3G1Y6, Canada; patrik.abdelnour@mail.mcgill.ca (P.A.); daniel.chow@mail.mcgill.ca (D.R.C.); 3 Montreal Glaucoma Institute and Bellevue Ophthalmology Clinics, Department of Ophthalmology, University of Montreal, Montreal, QC H3T 1J4, Canada

**Keywords:** machine learning, glaucoma, visual fields

## Abstract

Visual field (VF) testing is crucial for the management of glaucoma. However, the process is often hindered by technician shortages and reliability issues. In this study, we leveraged machine learning to predict pattern standard deviation (PSD) using clinical inputs. This machine learning retrospective study used publicly accessible data from 743 eyes (541 glaucoma and 202 non-glaucoma controls). An automated neural network (ANN) model was trained using seven clinical input features: mean retinal nerve fiber layer (RNFL), IOP, patient age, CCT, glaucoma diagnosis, study protocol, and laterality. The ANN demonstrated efficient training across 1000 epochs, with consistent error reduction in training and test sets. Mean RMSEs were 1.67 ± 0.05 for training, and 2.27 ± 0.27 for testing. The *r* was 0.89 ± 0.01 for training, and 0.81 ± 0.04 for testing, indicating strong predictive accuracy with minimal overfitting. The LOFO analysis revealed that the primary contributors to PSD prediction were RNFL, CCT, IOP, glaucoma status, study protocol, and age, listed in order of significance. Our neural network successfully predicted PSD from RNFL and clinical data with strong performance metrics, in addition to demonstrating construct validity. This work demonstrates that neural networks hold the potential to predict or even generate VF estimations based solely on RNFL and clinical inputs.

## 1. Introduction

Glaucoma is the second most prevalent eye condition and one of the leading causes of irreversible blindness globally [1,2]. This group of disorders present with chronic progressive optic neuropathy in addition to characteristic morphological changes at the optic nerve head and in the retinal nerve fiber layer (RNFL) [3]. Although optic neuropathy in glaucoma cannot be halted, progression of vision loss can be attenuated with proper treatment aimed at decreasing intraocular pressure (IOP) [3]. While the management of glaucoma focuses on controlling IOP, lifelong assessment of disease progression hinges on monitoring visual field (VF) measurements, including mean deviation (MD) and pattern standard deviation (PSD) [4]. Clinically, MD is more often used to monitor overall depression in glaucoma, while PSD monitors focal defects [3]. Unfortunately, VF testing to obtain these VF measurements involves several challenges, namely severe technician shortages and reliability concerns due to the subjective nature of the assessment [5,6]. Studies have shown that a large portion of patients had unreliable VF test results, with unreliability rates as high as 35% in patients with ocular hypertension [7,8,9]. Other barriers to reliable VF testing are retinal pathologies worsening VFs, rendering the interpretation of VF measurements more challenging [3]. Moreover, predictions for 2035 highlight that the pressing workforce concern may not be ophthalmologist supply, but rather support staff, such as technicians [10]. Estimates also suggest that the direct healthcare cost of treating primary open-angle glaucoma in Canada is 300 million (Canadian dollars) per year and increasing, with expenses primarily driven by VF-based monitoring [11]. The demand for more reliable and cost-effective assessments, as well as decreased reliance on technicians, is clear.

Some groups have attempted to solve the issues of technician dependency and subjective assessment through promising concepts such as self-administered testing and visually evoked potentials, but none have yet proved appropriate or ready for widespread use to date [12,13]. While machine learning has been used for glaucoma detection based on VF tests, little effort has been focused on applying this technology to predict VF measurements themselves [14,15,16,17]. Many attempts at applying deep learning to estimate VF values using optical coherence tomography (OCT) have been made, but little to no investigations included clinical data in their models, while they also rarely and less accurately predict pattern standard deviation (PSD) [18,19,20]. Although current evidence on estimating VF measurements using machine learning is promising, there remains a need to provide accurate estimates for PSD, as it is extremely relevant in the context of glaucoma detection and monitoring [3].

Considering the need for PSD testing in glaucoma that is less technician-reliant and subjective, we aimed to leverage machine learning to solve this issue. In this study, we used an automated network model (ANN) to predict PSD using clinical data and OCT RNFL.

## 2. Materials and Methods

### 2.1. Participants

This was a machine learning retrospective study using data from two publicly accessible repositories: (1) from a study conducted at Dankook University Hospital and Gyeongsang National University Hospital (Dankook-GNU) [21], and (2) the GRAPE [22] data set, including 499 and 263 eyes, respectively. For the Dankook-GNU study, eyes were included if they met the following inclusion criteria: best-corrected visual acuity ≥20/40 and normal anterior segment on slit-lamp examination. To be included in the glaucomatous eyes group, as opposed to the non-glaucomatous control eyes, a glaucoma diagnosis (primary open-angle or normal-tension) was also needed. Although the dataset contained more glaucoma than control eyes, we retained all available cases to maximize statistical power, as matching was not required for model training or continuous outcome prediction. In regression-based neural networks, the goal is to learn relationships between predictors and the target variable rather than estimate group prevalence, and thus moderate imbalances do not bias model performance in the same way they might for binary classification tasks. Diagnoses were rendered by the principal or co-investigator and required characteristic optic-disc structural damage accompanied by reproducible glaucomatous visual-field loss, defined as at least one of the following on automated perimetry: a glaucoma hemifield test outside normal limits, a PSD probability < 5%, or a cluster of ≥3 points (all *p* < 5%, with ≥1 point *p* < 1%) in a single hemifield. Exclusion criteria consisted of history of ocular inflammation or trauma; and the presence of concurrent retinal disease, optic nerve disease other than glaucoma, or a brain disorder that could influence the visual field results [21]. For the GRAPE study, only glaucomatous eyes were included (either open-angle [*n* = 139] or angle-closure glaucoma [*n* = 124]). Diagnosis followed the American Academy of Ophthalmology Preferred Practice Pattern guidelines requiring a comprehensive review of medical history in addition to concordant findings on functional assessment (VF), structural imaging (CFP or OCT or both), IOP, central corneal thickness (CCT), and gonioscopic evaluation of the anterior chamber angle. All cases were first diagnosed by senior outpatient clinicians and then confirmed by three glaucoma specialists. Eyes were excluded if they had other optic-nerve diseases, severe retinal pathology, significant media opacity, prior glaucoma surgery, or were under 18 years old [22].

### 2.2. Data

The clinical factors extracted from both the Dankook-GNU and GRAPE studies were PSD, RNFL, CCT, laterality, initial untreated IOP, glaucoma diagnosis, and age from the patient’s initial visit and assessment. These datasets were selected because they are publicly accessible, well-annotated, and provide sufficient sample size to enable robust model training and testing. Both the Dankook-GNU and GRAPE datasets included the necessary clinical inputs, OCT-derived parameters, and visual field measurements required for our analysis. Importantly, they also contained both glaucomatous and non-glaucomatous eyes, allowing the model to learn from a heterogeneous population and capture clinically meaningful differences. In addition, the datasets were derived from independent cohorts in different institutions, which adds diversity to the data and helps reduce single-center bias. Together, these factors made the Dankook-GNU and GRAPE datasets well-suited for the present study.

The Dankook-GNU study collected data from patients seen at Dankook University Hospital and Gyeongsang National University Hospital between 2012 and 2015. Patients underwent standardized ophthalmic evaluations including best-corrected visual acuity, slit-lamp examination, Goldmann applanation tonometry, CCT measurement, dilated fundus photography (Canon, Tokyo, Japan), spectral-domain-OCT using Spectralis (Heidelberg Engineering GmbH, Heidelberg, Germany), and VF testing using the 30-2 SITA-Standard protocol on a Humphrey Field Analyzer 740 (Carl Zeiss Meditec Inc., Dublin, CA, USA).

The GRAPE dataset was collected between 2015 and 2022 at the Eye Center of the Second Affiliated Hospital of Zhejiang University. Patients received comprehensive glaucoma evaluations, including IOP and CCT measurements using a non-contact tonometer NT-530P (NIDEK, Gamamori, Japan), peripapillary RNFL thickness measurement with CIRRUS HD-OCT 5000 (Carl Zeiss Meditec, Dublin, CA, USA), and fundus photography using a 50° field of view centered at macula by the TRC-NW8 Fundus Camera (TOPCON, Tokyo, Japan), CR-2 PLUS AF Digital Retinal Camera (CANON, Tokyo, Japan) and CR-2 AF Digital Retinal Camera (CANON, Tokyo, Japan) without pupil dilation. Visual fields were obtained using the G1 program on the Octopus 900 perimeter (Haag-Streit, Köniz, Switzerland), and post-processing was performed to derive pattern standard deviation (PSD) values from the raw threshold data. Poor-quality exams (false positives or negatives ≥30%) were excluded. Fundus images were quality-controlled, cropped to regions of interest centred on the optic disc, and manually annotated for optic disc and cup segmentation. Clinical data such as age, sex, and glaucoma type were extracted from electronic health records.

### 2.3. Sample Size Rational

With seven candidate predictors, our estimated minimum required sample size was at least 105 eyes. This was obtained based on the commonly accepted guideline put forth by Peduzzi et al.: 10 to 15 observations per predictor to ensure model stability and avoid overfitting [23]. This guideline was also validated in 2007, reinforcing our confidence in the estimated required sample size [24]. The 762 extracted eyes from the Dankook-GNU and GRAPE studies far exceed the “105” threshold, suggesting our cohort is sufficiently powered.

### 2.4. Model Training and Validation

**Figure 1:**
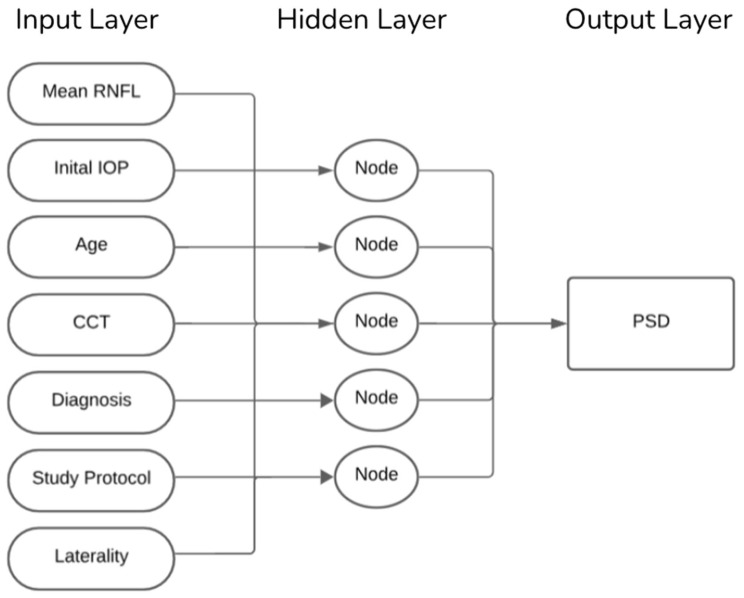
Network Architecture.

Eyes were randomly assigned, clustered by patients and stratified by diagnosis to a 90% training set and a 10% hold-out test set. Our ANN was trained using seven clinical input features: mean RNFL, IOP, patient age, CCT, glaucoma diagnosis (classified as non-glaucomatous eyes, primary open-angle glaucoma, or primary angle-closure glaucoma), study protocol (GRAPE vs. Dankook-GNU), and laterality (right vs. left eye). As illustrated in Figure 1, the model comprised a single hidden layer including five neurons and a linear output node trained using the scaled-conjugate-gradient algorithm. This architecture was selected to balance complexity and interpretability, ensuring that the network could capture non-linear relationships between features without overfitting, given the dataset size. Training stopped after reaching 1000 epochs. Weights were initialized randomly at the start of each training run, and model parameters were updated iteratively to minimize the mean squared error between predicted and observed PSD values. A validation set derived from the training data was used internally to monitor convergence and prevent overfitting. To ensure stability and account for variability in training, each model configuration was trained 10 times. The performance metrics root mean squared error (RMSE) and Pearson’s *r* were averaged across these runs, and variability was reported as standard deviations. This repeated training strategy ensured that the final reported results were not due to chance weight initialization or a single favorable run but reflected robust and reproducible network behavior.

### 2.5. Statistical Analysis

Model performance was assessed using the RMSE and Pearson correlation coefficient (*r*). Overall model strength was then evaluated using the average of the corresponding 10 performance metrics. All analyses were performed using MATLAB R2024b’s Statistics and Machine Learning Toolbox.

### 2.6. Leave One Factor out Analysis

To assess the contribution of each input factor in the model’s predictive performance, a leave-one-feature-out (LOFO) ablation analysis was performed. Following the overall performance evaluation (baseline), the model was then retrained iteratively with the same 10-run averaging approach, each time excluding a single factor. Changes in test set average RMSE and *r*, relative to the baseline metrics, were computed with paired two-tailed *t*-tests and used to quantify the importance of each factor. A two-tailed paired *t*-test was selected to allow for the possibility that model performance could be either improved or worsened when a given feature was removed, rather than assuming a directional effect. All *p*-values < 0.05 were considered statistically significant in this investigation. This method provides a model-specific and comprehensive estimate of factor importance by directly measuring the impact of individual variables on predictive outcomes [25]. In addition, this analysis also provides construct validity by demonstrating which clinically relevant or irrelevant factor inputs contribute significantly to model predictions [26]. Performing the LOFO method is in keeping with well-established practices in model interpretation and local feature attribution, which aim to produce transparent and meaningful explanations of model behavior [26,27].

## 3. Results

**Figure 2:**
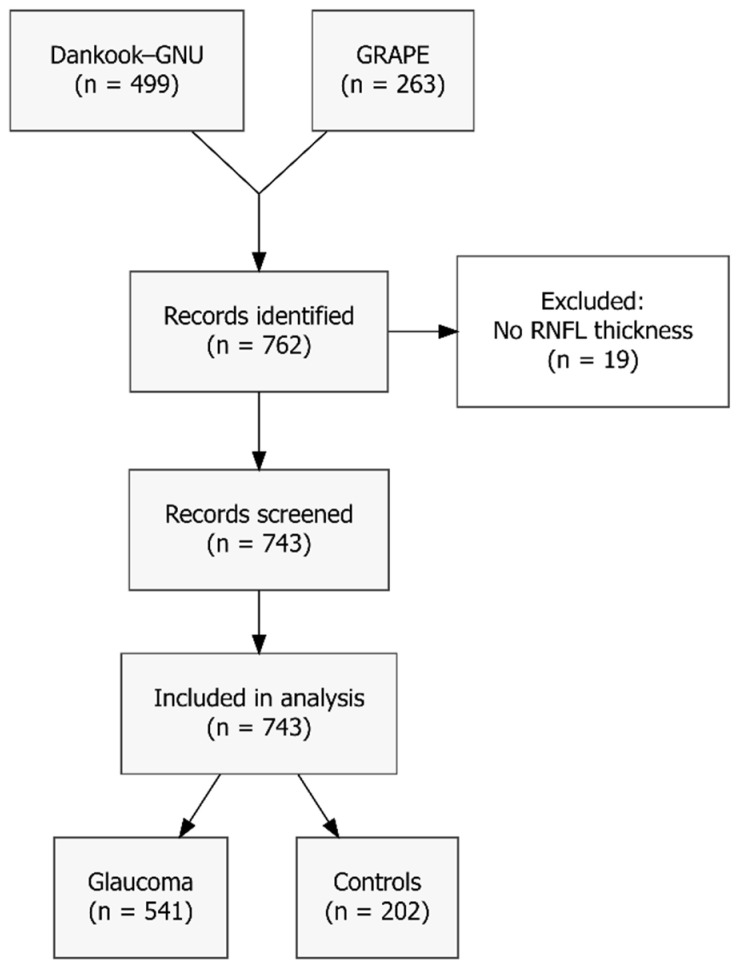
The Flow Diagram illustrates the recruitment of 743 eyes from the Dankook-GNU and GRAPE studies.

Although 762 eyes were extracted from the Dankook-GNU and GRAPE studies, 19 eyes (2.5%) were excluded as they lacked global peripapillary RNFL thickness measurements. The resulting sample consists of 743 eyes (541 with glaucoma and 202 controls), as illustrated in Figure 2. Mean age ± standard deviation (SD) was 51.7 ± 16.9 years; mean IOP was 19.8 ± 7.7 mm Hg, mean CCT was 538.6 ± 33.6 µm and mean global RNFL thickness was 81.6 ± 23.1 µm. Overall mean PSD was 5.91 ± 3.65 dB, with mean PSD in glaucomatous eyes being even higher (7.33 ± 3.32 dB). Complete baseline characteristics are presented in Table 1.

As presented in Table 2, our ANN achieved strong performance metrics. Overall, the model obtained an RMSE of 1.67 ± 0.05 and an *r* of 0.89 ± 0.01 for the training set, as well as an RMSE of 2.27 ± 0.27 and an *r* of 0.81 ± 0.04 for the testing set. The best out of the 10 networks obtained an RMSE of 1.69 and an *r* of 0.89 for the training set, as well as an RMSE of 2.05 and an *r* of 0.83 for the testing set.

The results of the LOFO analysis presented in Table 3 revealed that all the factors used to train our ANN had strong predictive importance except for laterality. Factors decreased in predictive importance in the following order: RNFL (ΔRMSE 4.12 ± 1.10, *p* = 0.001), CCT (ΔRMSE 3.90 ± 0.76, *p* < 0.001), IOP (ΔRMSE 3.75 ± 0.95, *p* < 0.001), glaucoma status (ΔRMSE 3.39 ± 0.54, *p* < 0.001), study protocol (ΔRMSE 3.17 ± 0.60, *p* < 0.001), age (ΔRMSE 2.69 ± 0.31, *p* = 0.004), and laterality (ΔRMSE 2.18 ± 0.26, *p* = 0.391). This order of importance was also consistent across the Δ*r* values for each factor.

**Figure 3:**
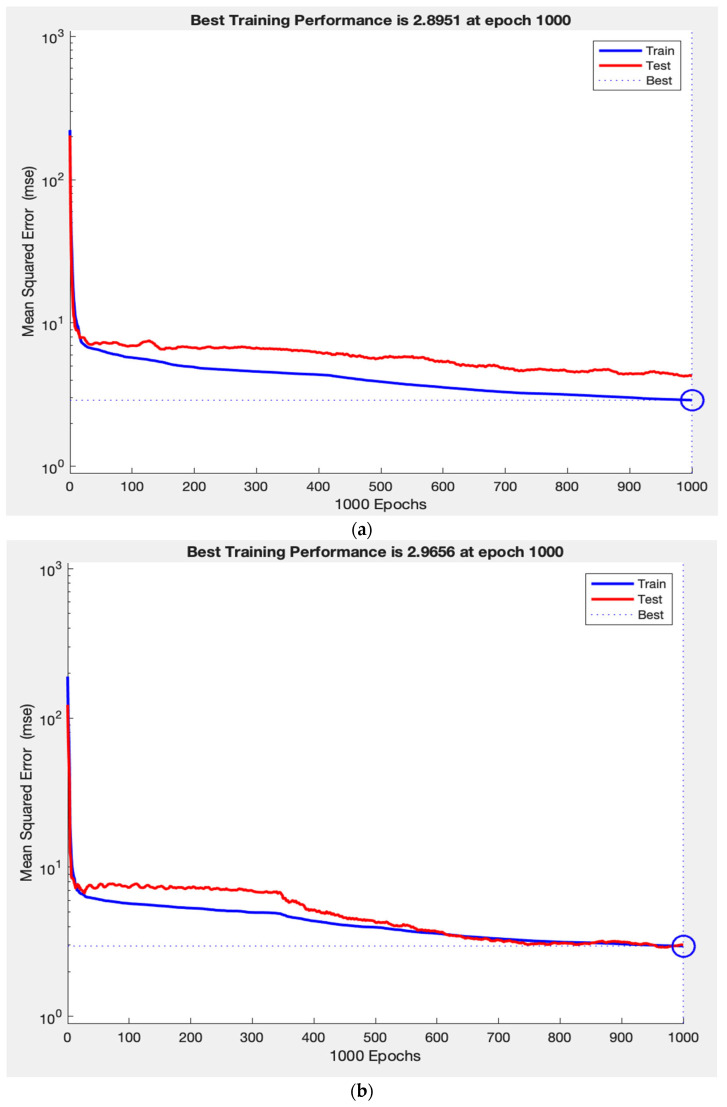

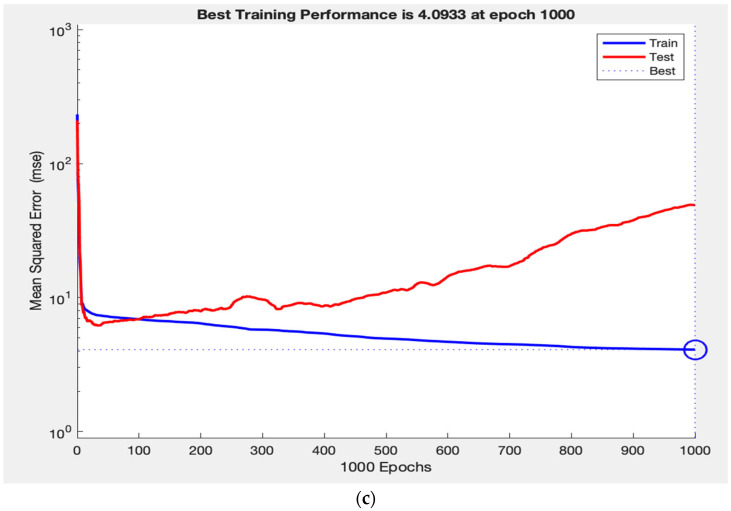
Training- and validation-loss curves for the neural-network regressors. Mean-squared error (log_10_ scale) vs. epoch for training (blue) and test sets (red); (**a**) Baseline model (seven predictors). Training and test curves converge and remain stable thereafter, indicating stable generalization and absence of over-fitting. (**b**) Model with laterality removed. Both curves converge more tightly, and the validation plateau drops slightly, mirroring the modest RMSE improvement when laterality is removed. This pattern indicates that our model identified the low predictive value of laterality, further highlighting the absence of over-fitting. (**c**) Model with mean RNFL removed. The training curve continues to decrease while the test curve rises, indicating overfitting. This pattern reflects a substantial loss in generalization performance when mean RNFL is excluded from the model inputs, demonstrating its importance as a predictive factor.

Throughout training, the network’s performance consistently increased on both the training and test sets, indicating that the model is learning a generalizable mapping, rather than simply memorizing the data. This is demonstrated in Figure 3a, where the training and test loss curves converge and stabilize, reflecting strong generalization. In contrast, Figure 3c shows that removing RNFL caused the test loss curve to diverge abruptly while the training loss curve continues to decline, demonstrating a typical pattern of overfitting. Finally, Figure 3b illustrates how removing laterality resulted in slightly improved test performance and tighter curve convergence, indicating that laterality contributes minimally to the model’s predictive capacity. This pattern supports the construct validity of our ANN, as the observed learning behaviours in predicting VF measurements reflect the intuitive clinical importance of RNFL, as well as the lack of clinical value.

## 4. Discussion

Given the rising prevalence of glaucoma, its link to blindness worldwide, and the importance of early detection and monitoring, addressing current limitations of VF testing is essential to guide clinical care [1,2]. The increasing demand for VF testing, in addition to the subjective nature and reliance on technicians, highlights the need for complementary VF data acquisition. It should also be noted that reliance on OCT alone, even when requiring technician acquisition, is still more resource-efficient than protocols necessitating both OCT and VF testing. VF testing is time-intensive, highly variable between patients, and often requires repeated trials under technician supervision. In contrast, OCT imaging is relatively rapid, objective, and less burdensome on staff time. Thus, a model leveraging OCT with clinical variables offers a practical advantage by reducing technician workload and patient testing burden compared to approaches dependent on both OCT and VF acquisition. To our knowledge, this is the first study to leverage machine learning to predict PSD using RNFL and clinical data in glaucoma patients. Our ANN successfully predicted PSD from RNFL and clinical data with strong performance metrics. The primary clinical contributors to PSD prediction were mean RNFL, diagnosis, CCT, IOP, and age, in decreasing order of impact. The findings of this study suggest PSD can be accurately estimated in glaucoma patients without having to perform VF testing, adding new options for obtaining VF measurements.

The ANN model employed in this investigation predicted PSD with stronger performance metrics than those observed in similar studies. When applied to the test dataset, our model had an RMSE of 2.27 dB and an R of 0.81. Furthermore, comparing the test set RMSE of 2.27 dB to the 1.67 dB for the training set suggests the error on both sets is reasonably low and not diverging, highlighting consistent learning and minimal overfitting. A similar study tested five separate regression models predicting VF measures from RNFL, ganglion cell inner plexiform layers, and macular thickness, with the XGboost regressor being the strongest model for estimating PSD measurements and showing an R of 0.76 as well as an RMSE of 2.34 [28]. Although the performance metrics for this regressor are strong, they are weaker than those seen in our neural network model. Christopher et al. [19] conducted another investigation leveraging a deep learning model to differentiate between eyes with and without glaucomatous visual field damage and predicting the severity from OCT. When using their best model, which is based on RNFL enface images, they found an R^2^ of 0.61 when estimating PSD [19]. Although an R^2^ of 0.61 is typically acceptable, it is less than the 0.65 exhibited by our model. Unlike many prior studies that rely on ganglion cell layer (GCL) or macular thickness metrics, this study uses only RNFL measurements along with clinical data. This is particularly advantageous because in patients with concurrent retinal pathologies (such as diabetic macular edema or epiretinal membranes) that may confound GCL or macular analyses, RNFL remains relatively preserved as a marker of glaucomatous damage. Thus, this approach allows for more accurate identification and staging of visual field defects specifically attributable to glaucoma, even in the presence of macular disease. Moreover, Kim et al. [21] used a deep-learning model to predict MD and PSD from Bruch’s Membrane Opening-Minimum Rim Width and RNFL. They observed ranges of Pearson’s correlation coefficients between 0.74–0.82 (min-max) and ranges of MAE between 1.6–2.0 dB when predicting PSD. The Pearson’s correlation coefficient of 0.83 observed in our study is above the range of R values observed by Kim et al., insinuating that our model accounted for a higher percentage of the variance [21]. Although difficult to directly compare with existing models due to differences in study design and evaluation metrics, the evidence discussed above underscores the strong performance of our ANN to predict PSD. This strong performance may be attributed to our novel inclusion of clinical data in addition to RNFL in our model. Importantly, when considering RNFL as a standalone predictor, performance metrics were consistently lower than those achieved by the integrated ANN model. This highlights that while RNFL is valuable, it does not fully capture the multifactorial determinants of PSD, and the addition of clinical variables substantially enhances predictive accuracy. Thus, our model not only leverages OCT-derived structural data but also reduces reliance on OCT alone by contextualizing it within broader patient-level factors. Only a limited number of studies have attempted to estimate PSD using machine learning, especially in comparison to the large body of work focused on estimating MD. As interest in estimating PSD grows, future studies may report models that match or even surpass our performance.

Although multifactorial, the high performance of our model to predict PSD can be attributed in part to our inclusion of clinical data in addition to OCT measurements. Given the clinical relevance and well-established role of OCT measurements in glaucoma models, it is not surprising that RNFL emerged as our main contributor in the LOFO analysis [18,19,20,21,28]. In addition, CCT was the second most important contributor, adding to the growing literature supporting thin CCT as a biomarker for functionally glaucomatous damage [29]. Interestingly, myopes tend to have both thinner CCT and RNFL, which were factors identified by the network as key predictors of PSD. This suggests that incorporating myopia status, whether defined by axial length or refractive error, could potentially improve network performance. Overall, the LOFO analysis revealed that factors decreased in predictive importance according to the following order: RNFL, CCT, IOP, glaucoma status, study protocol and age. It is consistent with clinical understanding that glaucoma diagnosis was an important contributor, as distinguishing between non-glaucomatous, POAG, and PACG eyes added predictive value for PSD estimation. The predictive importance of the study protocol reflects differences in OCT and VF acquisition between the GRAPE and Dankook-GNU datasets. While RNFL emerged as the most influential contributor in the LOFO analysis, the ANN’s superior predictive performance arose from its ability to synergistically integrate RNFL with other clinical features. Conversely, the lack of laterality’s predictive value aligns with its lack of clinical relevance in VF outcomes. Collectively, these findings support the construct validity of our ANN by demonstrating alignment between model-driven feature attribution and known clinical relevance. Furthermore, the alignment of LOFO-supported predictors with established clinical factors affecting VF measurements suggests our ANN could identify important variables related to VF prediction, providing reassurance that the model is learning clinically meaningful patterns and associations, rather than memorizing data.

Rather than considering these tools in isolation, there is potential value in applying them sequentially. The La Roche Glaucoma Risk Calculator, which leverages age, IOP, and CCT, could be effectively used as an initial screening tool to identify individuals at higher risk who may benefit from more advanced assessments such as ANN-based PSD prediction [30]. This is particularly valuable for elderly populations or in regions with limited access to OCT and VF technologies, where resource-efficient screening is crucial. Our LOFO analysis reinforces the relevance of the La Roche calculator, as it similarly identified CCT, IOP, and age among the most important clinical predictors of glaucoma-related functional damage. Together, this suggests that integrating risk calculators with ANN models could create a synergistic pathway: inexpensive broad screening followed by precision modelling for those identified as high risk.

The main limitation of our study is the sample size of 743 eyes. Although appropriately powered, other similar investigations did boast larger samples [19,20]. Secondly, the datasets used in this study were not matched for ethnic background or age. All patients were from East Asia, which may limit the generalizability of our findings to more diverse populations. Prior work by Girkin et al. has shown that optic nerve head morphology and RNFL thickness vary with both age and ethnicity [31]. Thus, the lack of diversity and age matching in our datasets could have influenced the variation in RNFL inputs to our model and should be considered when interpreting the external validity of its predictions in populations outside East Asia and across different age groups. A limitation of this approach is that it relies on RNFL thickness to predict functional loss. As a result, patients with retrobulbar neuro-ophthalmic conditions (such as compressive optic neuropathies or optic neuritis) may not exhibit RNFL thinning despite significant visual field defects. Consequently, neuro-ophthalmic mimickers of glaucoma could be missed by this model, underscoring the need for comprehensive clinical evaluation to rule out alternative causes of visual field loss. Future work should focus on validating this ANN with real-world data. To ensure clinical applicability and external validity, such efforts should ideally involve prospective data collection across multiple centers and countries. Multicentered, multinational datasets would capture greater variability in patient demographics, disease severity, and imaging platforms, thereby improving the model’s generalizability and reducing the risk of bias associated with single-center retrospective analyses. Incorporating such diverse real-world data will be an essential step toward establishing the robustness and utility of this approach in clinical practice.

## 5. Conclusions

This study demonstrated the potential of an ANN model to accurately predict PSD in glaucoma patients using RNFL and clinical data. Not only did our model achieve strong performance metrics and demonstrate construct validity, but it also outperformed previous models relying solely on imaging inputs, such as RNFL, macular thickness, and ganglion cell inner plexiform layer. Moreover, we observed that RNFL as well as clinical inputs, such as CCT and IOP, were strong contributors to estimating PSD. The significant role CCT plays in PSD prediction supports the growing evidence underlying thin CCT as a potential biomarker for functional glaucomatous damage. These findings open new avenues for obtaining practical and objective VF estimations in glaucoma patients, while reducing dependence on labour-intensive technician involvement.

## Figures and Tables

**Table 1:** Baseline characteristics of both patient populations.

Characteristics	All Eyes (*n* = 743)	Glaucomatous Eyes (*n* = 541)	Non-Glaucomatous Control (*n* = 202)
Sample provenance			
GRAPE Study (*n*; %)	244 (33)	244 (45)	0 (0)
Dankook-GNU Study (*n*; %)	499 (67)	297 (55)	202 (100)
Laterality			
Right eyes (*n*; %)	365 (49)	265 (49)	102 (51)
Left eyes (*n*; %)	378 (51)	276 (51)	100 (49)
Ocular characteristics			
RNFL (µm)	81.6 ± 23.1	72.8 ± 19.4	105.2 ± 14.0
CCT (µm)	538.6 ± 33.6	535.3 ± 32.6	547.4 ± 34.8
IOP (mm Hg)	19.8 ± 7.7	21.1 ± 8.4	16.4 ± 3.4
PSD (dB)	5.91 ± 3.65	7.3 ± 3.3	2.18 ± 0.85
Average age (yrs)	51.7 ± 16.9	51.8 ± 17.1	51.5 ± 16.5

**Table 2:** Network Performance.

Networks	Training		Testing	
	RMSE	*r*	RMSE	*r*
All networks	1.67 ± 0.05	0.89 ± 0.01	2.27 ± 0.27	0.81 ± 0.04
Best network	1.69	0.89	2.05	0.83

“All networks” refers to all 10 networks tested, with results expressed as mean ± SD. “Best network” refers to the network with the highest performance.

**Table 3:** Model LOFO analysis.

Factors Removed	RMSE	*p*	*r*	*p*
Base Network	2.26 ± 0.27		0.81 ± 0.04	
RNFL	4.12 ± 1.10	0.001	0.49 ± 0.10	<0.001
CCT	3.90 ± 0.76	<0.001	0.55 ± 0.08	<0.001
IOP	3.75 ± 0.95	<0.001	0.57 ± 0.10	<0.001
Glaucoma Status	3.39 ± 0.54	<0.001	0.59 ± 0.11	<0.001
Study protocol	3.17 ± 0.60	<0.001	0.64 ± 0.10	<0.001
Age	2.69 ± 0.31	0.004	0.70 ± 0.06	<0.001
Laterality	2.18 ± 0.26	0.391	0.78 ± 0.07	0.147

## Data Availability

The original contributions presented in this study are included in the article. Further inquiries can be directed to the corresponding author.

## Abbreviations

The following abbreviations are used in this manuscript:

VF	Visual Fields
RNFL	Retinal Nerve Fiber Layer
IOP	Intraocular Pressure
MD	Mean Deviation
PSD	Pattern Standard Deviation
ANN	Automated Neural Network
RMSE	Root Mean Squared Error
CCT	Central Corneal Thickness
LOFO	Leave-One-Factor-Out
GCL	Ganglion Cell Layer
OCT	Ocular Coherence Tomography
